# Granulomatous Liver Disease in Ataxia-Telangiectasia With the Hyper-IgM Phenotype: A Case Report

**DOI:** 10.3389/fped.2020.570330

**Published:** 2020-11-19

**Authors:** Aleksandra Szczawińska-Popłonyk, Lidia Ossowska, Katarzyna Jończyk-Potoczna

**Affiliations:** ^1^Department of Pediatric Pneumonology, Allergology and Clinical Immunology, Poznan University of Medical Sciences, Karol Jonscher University Hospital, Poznan, Poland; ^2^Department of Pediatric Radiology, Poznan University of Medical Sciences, Karol Jonscher University Hospital, Poznan, Poland

**Keywords:** primary immunodeficiency, hyper-IgM phenotype, granulomatous liver disease, hypersplenism, children

## Abstract

Ataxia-telangiectasia (A-T) is an autosomal recessive disorder characterized by neurodegeneration, combined immunodeficiency, and oculocutaneous telangiectasia. The hyper-IgM phenotype of A-T, correlating with a class-switch recombination defect, IgG and IgA deficiency, T helper and B cell lymphopenia, immune dysregulation, proinflammatory immune response, autoimmune disease, and a high risk of lymphomagenesis. Progressive liver disease is a hallmark of classical A-T with the hyper-IgM phenotype and manifests as non-alcoholic hepatic steatosis and fibrosis. We report a case of a 17-year-old male A-T patient, in whom a progressive granulomatous liver disease with portal hypertension, has led to massive splenomegaly and hypersplenism, metabolic liver insufficiency, bleeding from esophageal varices and pancytopenia. In this patient, an unusual severe disease course with a highly variable constellation of A-T symptomatology includes granulomatous skin, visceral, and internal organs disease with liver involvement. The liver disease is associated with the hyper-IgM immunophenotype and escalating neurodegeneration, creating a vicious circle of immune deficiency, permanent systemic inflammatory response, and organ-specific immunopathology.

## Background

Ataxia-telangiectasia (A-T), also known as Louis-Bar or Boder-Sedgwick syndrome, is an autosomal recessive genomic instability syndrome, resulting from a mutation of the Ataxia-Telangiectasia Mutated (*ATM*) gene. The gene, localized to 11q22.3–23.1, encodes for a high-molecular-weight, predominantly a nuclear serine/threonine-protein kinase which is a member of the large phosphatidylinositol 3-kinase (PI3K)-related protein kinase (PIKK) family. The ATM kinase also plays many important cytoplasmic roles, phosphorylating numerous protein substrates and in mitochondrial respiration and energy metabolism. The enzyme is involved in maintaining the cell-cycle homeostasis and coordinates the cellular signaling pathways in response to DNA double-strand breaks (DSBs), genotoxic, and oxidative stress. The impact of the genetic background on the cellular nature of ataxia-telangiectasia is pleiotropic and the phenotype of the disease is complex and heterogeneous, varying among affected patients based on the severity of ATM mutations (1, 2). Versatile ATM roles determine a multisystem involvement in A-T, which is predominantly characterized by neurodegeneration with progressively debilitating cerebellar ataxia and muscle weakness, dysarthria, oculomotor apraxia, impaired coughing, and swallowing. Beyond striking neurological features, the extended A-T phenotype also includes dermatological manifestations, such as oculocutaneous telangiectasia and cutaneous granulomas, hormonal dysfunction, eg. growth retardation, insulin resistance, premature aging, infertility due to gonadal dysgenesis, as well as chronic airway and lung disease (3, 4). Combined immunodeficiency with immune dysregulation, a predisposition to lymphoid malignancies, and sensitivity to ionizing radiation are markers of chromosomal instability and impaired molecular mechanisms of the ATM kinase response to DNA damage. The ATM kinase plays an important role in the processes of lymphocyte development and acquirement of immunocompetence, which rely on its function of DNA double-strand break repair in course of the V(D)J recombination. The pathophysiology of successful lymphocyte antigen receptor genes rearrangement and class switch recombination (CSR) of immunoglobulin genes are therefore critically important ATM kinase functions (5–7). Disturbed B and T cell homeostasis and failure of immunosurveillance are significant contributors to the development of autoimmune and autoinflammatory disorders, organ-specific pathology, and lymphoproliferation in A-T affected children. Defective DNA repair, impaired oxidative metabolism, immunodeficiency with immune dysregulation are important contributing factors, playing a role in the pathophysiology of cutaneous, visceral, and deep-organ granulomatous disease in A-T. Whereas cutaneous granulomatous lesions have been extensively reported thus far (8–11), with live vaccine-derived rubella virus considered to be a unique contributor to the development of granulomatous skin disease (12, 13), but extra-dermal manifestations of granulomas in the internal organs, such as the lungs or liver (14, 15) and in other localizations (16) have been infrequently described in the A-T pediatric patients.

We herein report an unusual case of a 17-year-old boy affected by A-T who presents with extensive granulomatous liver disease, associated by cutaneous, visceral, and internal organs granulomatosis.

## Case Presentation

**Figure 1:**
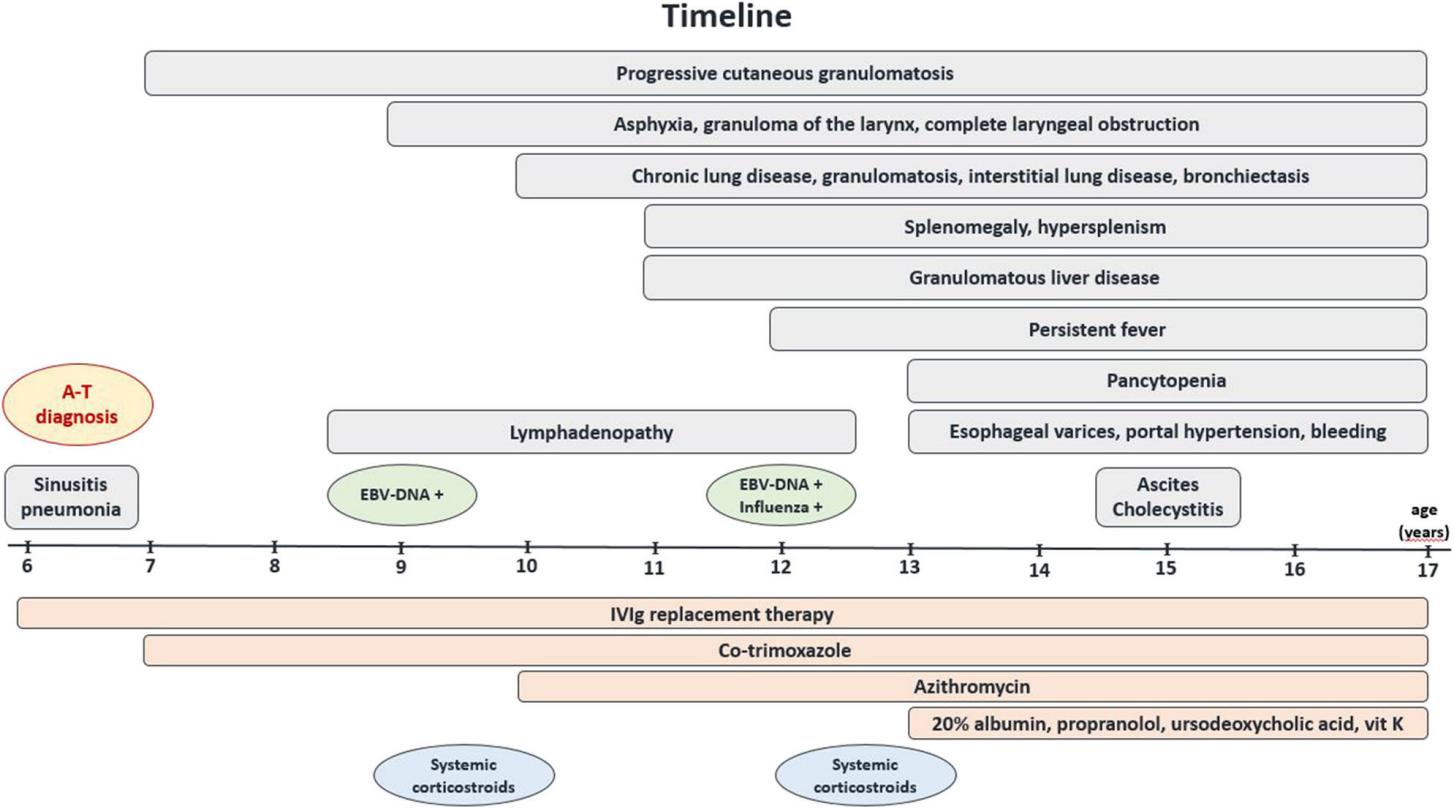
Timeline. Graphic display of the clinical course of the disease, complications, and the treatment used in the A–T patient with systemic granulomatosis.

A six-year-old boy was referred to our Department of Pediatric Pulmonology, Allergology, and Clinical Immunology because of pneumonia and sinusitis. Since the age of two, he presented with neurodevelopmental delay, progressive muscle weakness, disturbed gait, and because of these symptoms, he was systematically followed up by a pediatric neurology specialist who diagnosed the cerebellar form of cerebral palsy. At the age of three, he had severe varicella, without further neurological complications. By the age of 6, he suffered from recurrent upper and lower airway infections, frequently treated with antibiotics. He was born at term as the second child of non-consanguineous parents. The family history was contributory towards neoplastic and autoimmune diseases: his father suffered from psoriasis, and the mother was treated because of thyroid cancer. The striking clinical features of cerebellar ataxia with choreoathetotic movements, dystonia, dysarthria, and oculomotor apraxia were accompanied by oculocutaneous telangiectasia, palpable liver (+4 cm below the right costal margin), a granulomatous skin lesion of the left elbow, and malnutrition (weight and hight below the 3 percentile), along with laboratory tests showing immunodeficiency (lymphocytopenia, low IgG levels, a complete IgA deficiency), and a markedly elevated AFP level (192 mg/dL) implicated a preliminary diagnosis of A-T, which was further confirmed by a molecular ATM gene mutation analysis (7630-2A>C). The replacement therapy with monthly intravenous infusions of polyvalent immunoglobulins (IVIg) was then initiated and the child was followed up systematically (Figure 1). Two years later he developed cervical, submandibular, and mesenteric persistent lymphadenopathy, associated with an Epstein-Barr virus (EBV) infection (EBV-DNA PCR showed 1,500 viral DNA copies/mL), progressive disseminated cutaneous and visceral granulomatosis, and an episode of asphyxia, that was caused by a granuloma of the larynx leading to its complete obstruction at a distance of 1.8 cm that required an emergency tracheostomy. During the revision of tracheostomy, the laryngeal tumor was biopsied and immunohistochemistry showed infiltrations with predominating T CD8+ cells (CD4+:CD8+ ratio < <1) and histiocytes, with 30% of cells showing expression of the nuclear proliferation marker Ki-67. TdT (Terminal deoxynucleotidyl transferase), CD34 (hematopoietic progenitor cell antigen), CALLA (common acute lymphoblastic leukemia antigen), CD1a (antigen-presenting molecule on Langerhans cells and dendritic cells), LMP EBV (latent membrane protein, EBV oncoprotein), CD30 TNFRSF8 (TNF receptor superfamily member 8, contributing to lymphomagenesis), MPO (neutrophil myeloperoxidase) were not detected. Since that time, the patient manifested chronic airway and lung disease with bronchiectasis, interstitial involvement, and formation of granulomas. Three years later, a reactivation of EBV and simultaneous influenza A infection triggered recurrent fever episodes. As oral feeding was severely disturbed due to laryngeal tumor and tracheostomy, impaired coordination of breathing and swallowing, as well as massive fibrotic pharyngeal and palatal strands, altering the anatomy of the upper airways and the oral cavity, and failure to thrive, gastrostomy was proposed to enable safe and effective alimentary supply, but patient's parents did not agree for the procedure.

### Chronic Liver Disease

**Figure 2:**
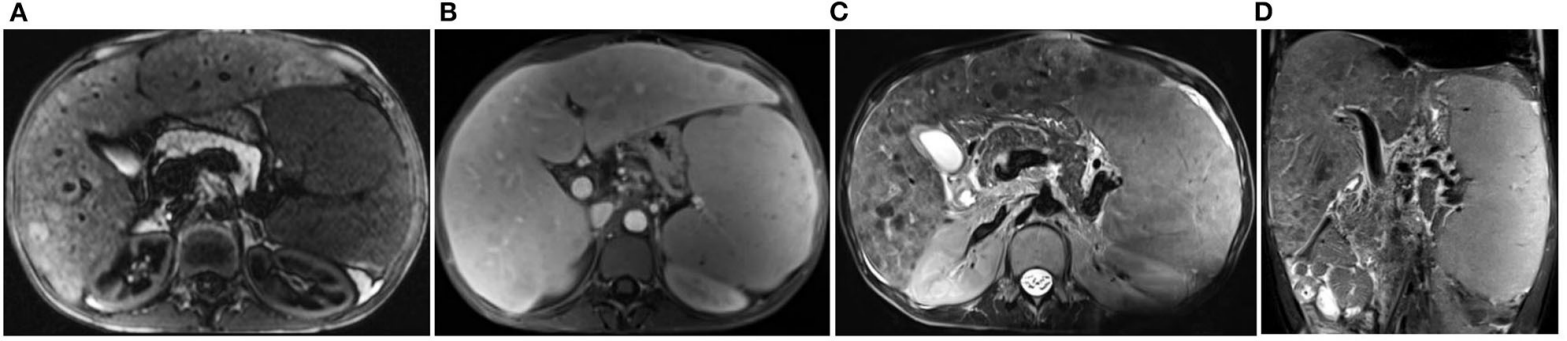
Magnetic resonance imaging of the abdominal cavity. Markedly enlarged spleen (19.1 × 8.2 cm) with irregular, nodular reconstruction, with hypointense foci in T1 and T2 dependent images, 5–60 mm large, modeling on vessels, and signal loss flow void foci in the central part of the spleen. The liver enlarged, the right lobe 18 cm, left lobe 14 cm, both lobes showing irregular nodular reconstruction, in T1 image hyperintense, in T2 image hypointense. All images show different phases of one MRI examination in the patient aged 17. **(A)** Transverse view, T1-weighted gradient-echo HASTE (Turbo spin-echo) sequence, oppose-phase imaging. **(B)** Transverse view, 3D starVIBE T1-weighted gradient-echo sequence FS (fat-suppressed), contrast protocol. **(C)** Transverse view, T2 image with SPC pulse sequence. **(D)** Coronal view, T2 image HASTE FS sequence.

Since the age of eleven, progressive chronic liver disease was observed, with splenomegaly and hypersplenism, pancytopenia, a markedly increased gamma-glutamyl transpeptidase (γGT) activity, the most sensitive marker of liver disease in A-T, hypoproteinemia and hypoalbuminemia (Table 1). Further rapid deterioration and complications with an episode of ascites and cholecystitis as well as the development of portal hypertension and esophageal varices (II/III grade in the endoscopic examination) were leading to two episodes of life-threatening massive bleeding at the age of sixteen and seventeen. The abdominal MRI imaging showed features of hepatosplenomegaly, with nodular regenerative hyperplasia of the liver and multiple granulomas in the spleen (Figure 2). The unstable patient's condition and chronic pancytopenia with recurrent very low numbers of thrombocytes (45–80 × 10^3^) rendered liver biopsy unreasonable. Due to the altered anatomical and functional conditions of the pharynx, larynx, and esophagus, endoscopic interventional banding of the esophageal varices was technically impossible, posing the need for the multidisciplinary approach and the further discussion on conservative management and/or surgical treatment.

**Table 1:** Laboratory workup of the patient (aged 17).

**Hematology**
WBC 2.41 × 10^3^, RBC 3.36 × 10^6^, MCV 89.0 fl, PLT 105 × 10^3^, lymphocytes 21.2% (510 cc/mcL), neutrophils 63.1% (1,520 cc/mcL)
APTT 51.7 s (25.9–36.6 s), prothrombin index 72.4% (80.0–120.0%), INR 1.37 (0.85–1.25), AT 54% (75.0–125.0%)
**Biochemistry**
CRP 0.22 mg/dL (<0.50 mg/dL), D-dimer 5.08 mg/L (<0.55)
Protein 4.57 g/dL (5.7–8.0 g/dL), Albumin 2,815 mg/dL (3,500–5,200 mg/dL)
Fibrinogen 124 mg/dL (180–350 mg/dL)
GOT 11 IU/L (<50 IU/L), GPT 30 IU/L (<50 IU/L), γGT 251 IU/L (2–42 IU/L)
LDH 214 IU/L (<248 IU/l)
Ferritin 115 mcg/L (15–250 mcg/L)
AFP 129.7 ng/mL (<7 ng/mL), CEA 0.75 ng/mL (<5.0 ng/mL)
**Immunology**
• Immunoglobulins 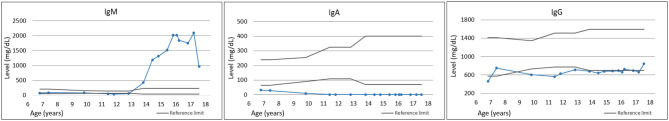
• Peripheral blood (PB) leukocyte immunophenotype
WBC 1,540 cc, lymphocytes CD45+/SSC low 20.0%, 305 cc (29–46%, 1,200–4,100 cc), B CD19+ 1.0%, 2 cc (7–22%, 100–820 cc), T CD3+ 77.0%, 264 cc (50–91%, 780–3,000 cc)
T helper CD3+CD4+ 52.0%, 176 cc (28–64%, 500–2,000 cc), T suppressor/cytotoxic CD3+CD8+ 19.0%, 65 cc (12–40%, 200–1,200 cc) CD4+/CD8+ 2.68
NK CD3–CD45+CD16+CD56+ 10.0%, 35 cc (5–49%, 100–1,200 cc)
Recent thymic emigrants CD3+CD4+CD45RA+CD31+: 1.1%, 2 cc (7–90%, 50–2,400 cc)
Naïve T helper CD3+CD4+CD45RA+CD27+ 0.7%, 1 cc (16–90%, 100–2,300 cc)
Central memory T helper CD3+CD4+CD45RA–CD27+ 40.5%, 71 cc (18–95%, 180–1,100 cc)
Effector memory T helper CD3+CD4+CD45RA–CD27– 58.5%, 103 cc (1–23%, 13–220 cc)
Terminally differentiated memory T helper CD3+CD4+CD45RA+CD27– 0.2%, 0 cc (0–7%, 0.0–0.7 cc)
Follicular CXCR5+ T helper CD3+CD4+CD45RO+CD185+ 4.7%, 8 cc (5–56%, 24–190 cc)
Regulatory T helper CD3+CD4+CD25++CD127– 3.2%, 6 cc (4–17%, 25–180 cc)
Naïve T suppressor/cytotoxic CD3+CD8+CD27+CD197+ 41.8%, 27 cc (6–90%, 16–1,000 cc)
Central memory T suppressor/cytotoxic CD3+CD8+CD45RA–CD27+CD197+ 10.4%, 7 cc (1–20%, 4–120 cc)
Effector memory T suppressor/cytotoxic CD3+CD8+CD45RA–CD27–CD197– 6.9%, 5 cc (14–98%, 40–640 cc)
Terminally differentiated T suppressor/cytotoxic CD3+CD8+CD45RA+CD27–CD197– 5.0%, 3 cc (7–53%, 25–280 cc)
Comment: lymphopenia, almost total lack of B cells, low T CD4+ and CD8+ cells, almost total lack of naïve T helper cells and recent thymic emigrants, with predominance of T helper effector cells, low effector and terminally differentiated T suppressor/cytotoxic cells
**Microbiology**
• EBV-DNA 135 copies/mL, HBV, HCV, CMV RT-PCR in PB negative
• VZV, *Enterovirus, Adenovirus, Parechovirus*, hPVB19, HSV1.2, HHV6.7 RT-PCR in PB negative
• *Influenza virus* A, AH1N1, B, *Coronavirus* NL63, 229E, OC43, HKU1, *Parainfluenza virus* 1,2,3,4, *Metapneumovirus* A,B, *Bocavirus*, RSV A,B, *Rhinovirus, Adenovirus, Enterovirus, Parechovirus, Mycoplasma pneumoniae, Chlamydophila pneumoniae, Streptococcus pneumoniae, Staphylococcus aureus* RT-PCR in nasopharyngeal aspirate negative
• Tracheal aspirate culture: negative
• *Pneumocystis jiroveci*-DNA in tracheal aspirate negative
• *Mycobacterium tuberculosis* (Mtb) in tracheal aspirate, Mtb-DNA negative
• *Borrelia burgdorferi* IgM negative
• *Bartonella henselae*-DNA, *Toxoplasma gondii*-DNA, *Toxocara canis*-DNA in PB negative
• HIV-RNA negative
• Galactomannan (*Aspergillus* antigen) in PB negative
**Histology**
• Bone marrow aspiration: normal cellularity; no features of malignant transformation; cytogenetics: deletions (5q–, 7q–, and 20q–) not found
• Skin biopsy: atypical granuloma annulare
• Biopsy of the larynx: infiltrations with reactive T CD8+ lymphocytes and CD68+ histiocytes, CD4+:CD8+ ratio < <1; 30% cells express Ki67; TdT, CD34, CALLA, CD1a, LMP EBV, CD30 TNFRSF8, and MPO not detected

The immunodiagnostic workup was systematically repeated and with time, a characteristic progressive hyper-IgM phenotype reflecting defective immunoglobulin class-switching, accompanied by complete IgA deficiency were observed, with a profound T-cell lymphopenia and an almost total lack of B cells in peripheral blood lymph cell immunophenotyping (Table 1). The clinical phenotype with chronic lung disease, lymphadenopathy, and febrile episodes implicated an extensive diagnostics towards viral, bacterial, and mycotic infectious pathogens, which revealed colonies of *Serratia marcescens, Streptococcus pneumoniae*, and *Pseudomonas aeruginosa* in the tracheostomy aspirate cultures.

Whereas pancytopenia may not only directly result from hepatosplenomegaly and hypersplenism, bone marrow was repeatedly biopsied and histological examination carried out to evaluate the maturation of major hematopoietic lines and to exclude malignant transformation and also for cytogenetic examination to exclude myelodysplastic syndrome (MDS)-related cytopenia.

### Therapy and Prognosis

At the diagnosis of A-T and immunodeficiency, at the age of six, substitution therapy with monthly high-dose IVIg was introduced and prophylaxis with co-trimoxazole was initiated. Three years later, regarding clinical and MRI features of chronic structural and interstitial lung disease, and the presence of bacterial superinfection in the tracheal aspirates, the course of antibiotic therapy with ceftazidime and amikacin, followed by alternate-day azithromycin antiinfective prophylaxis were administered. Two-month corticosteroid therapy with methylprednisolone in an initial dose of 2 mg/kg and its gradual decrease with the final alternate-day base was introduced twice and a moderate, transient favorable response was achieved, with a decrease of lymphadenopathy and a partial remission of cutaneous granulomas. Due to the chronic liver insufficiency with portal hypertension, propranolol, spironolactone, and ursodeoxycholic acid were given on a long-term basis, and blood-derived preparations, somatostatin, terlipressin, tranexamic acid, and vitamin K interim (Figure 1). Banding of esophageal varices to minimize the risk of bleeding and percutaneous endoscopic gastrostomy (PEG) placement to improve the patient's nutrition were successfully carried out. At the age of seventeen, all these treatment modalities have led to the stability of the patient's clinical condition and metabolic liver function. The opinion of the joint multidisciplinary consultations of pediatric specialists in immunology, hematooncology, pulmonology, gastroenterology, rheumatology, and infectious diseases was to continue the supportive treatment. The therapy with rituximab was also considered, but the unfavorable benefit-risk balance and a high risk of serious adverse effects, such as *Pneumocystis jiroveci* infection, cytopenia, and cytokine storm, led to disqualification from the treatment. The hematopoietic cell transplantation (HCT) was not recommended in this patient because of possible deleterious effects. Partial splenectomy was also discussed with surgeons and intensivists because of the potentially positive effect on pancytopenia, but the risk-benefit ratio of the surgery has been estimated to be very high.

## Discussion

Ataxia-telangiectasia is a devastating disease, which besides the neurodegeneration and motor disability, is characterized by multisystem involvement, combined immunodeficiency, predisposition to autoimmune and lymphoproliferative disorders, and a high risk of malignancy. A poorer prognosis and a reduced life expectancy have been shown in patients with the classical form of A-T and the hyper-IgM phenotype, IgG and IgA hypoimmunoglobulinemia, IgG2 subclass deficiency, and low numbers of circulating naïve T and B cells. Recurrent infections, chronic pulmonary disease, and the development of respiratory failure, and cancer are the most important contributors to a shortened survival (17). The hyper-IgM phenotype in A-T (A-T-HIGM) reflects a class-switch recombination defect (18–20), which was in the patient studied, characteristically correlated with hypoimmunoglobulinemia IgG, complete IgA deficiency, T cell lymphopenia, and a profound deficiency of recent thymic emigrant and naïve T helper cells. However, in contrast to other reported A-T-HIGM patients (5), in our patient, this phenotype developed in time, appeared at the age of fourteen, and followed but not preceded cerebellar ataxia in the early childhood.

Besides the symptoms of the neurodegenerative disorder, in the patient studied, the most striking feature of A-T was cutaneous and systemic granulomatosis, involving the larynx, the lungs, the joints, and the internal organs of the abdominal cavity, leading to massive hepatosplenomegaly with metabolic liver dysfunction, and hypersplenism. Combined immunodeficiency in our patient with A-T-HIGM is therefore not only a result of a low T cell thymic neogenesis and output, class-switch recombination defect, impaired T follicular lymph cell help to B cells, and a failure to mount effective B-cell response to the antigenic recall, but also results from the dysregulated liver and spleen functions, creating a vicious circle of immunodeficiency and organ-specific immunopathology. Whereas in older pediatric patients with classical A-T, a progressive non-alcoholic fatty liver disease, characterized by hypertransaminasemia and dyslipidemia is a frequent sequela (21, 22), a severe, life-threatening granulomatous disease in our patient belongs to a unique systemic granulomatosis phenotype, reflecting a complex immunopathology with the convergence of immunodeficiency, immune dysregulation, autoimmunity, and autoinflammation (23, 24).

In the patient studied, two episodes of EBV-DNA in blood were revealed, supporting other reports of increased susceptibility to EBV in A-T, resulting from thymic insufficiency and a defective generation of naïve T helper cells, leading to further impaired interferon-gamma (IFN-γ) production and a failure to antiviral defense mechanisms. Furthermore, ATM kinase participates in the control of chronic EBV infection, playing a role in the regulation of viral latency, contributing to the EBV-induced lymphomagenesis in A-T (25). However, the potential significance of the latent cycle of EBV in A-T and ATM deficiency in granulomatous disease has not been explored and remains a subject for further research.

It is worth noting, that systemic granulomatous disease with multiorgan involvement accompanying the liver disease has not been described in A-T children so far. Therefore, an unexpectedly severe granulomatous liver disease with portal hypertension, esophageal varices, and splenomegaly with hypersplenism in this patient sheds light on the role of ATM kinase in cellular metabolism and mitochondrial oxygen liver functions. A further multidisciplinary approach with the active participation of a pediatric gastroenterologist, surgeon, intensivist, and discussion on the perspectives of conservative, palliative, and surgical treatment, under the pediatric immunologist supervision, is currently of paramount importance.

## Concluding Remarks

The representative case report of a severe, progressive granulomatous disease with liver involvement, hyper-IgM phenotype, and thymic T cell neogenesis failure supports the hypothesis suggesting that A-T-HIGM patients represent a distinct clinical and immune phenotype with poor prognosis. From bench to bedside approach to the versatile role of ATM kinase in DNA double-strand breaks repair, oxygen metabolism and mitochondrial function, cell cycle regulation and proliferative checkpoint controlling is important in understanding the pathophysiology of the liver disease in A-T. Further studies on the liver-specific pathology, role of triggering a viral skewed immune response, immune dysregulation, and inflammatory disease, as well as genetic background with genotype-phenotype correlations, are required.

## Data Availability Statement

The data analyzed in this study is subject to the following licenses/restrictions: The raw data supporting the conclusions of this article will be made available by the authors, without due reservations, to any qualified researcher. Requests to access these datasets should be directed to Aleksandra Szczawińska-Popłonyk, aszczawinska@ump.edu.pl.

## Ethics Statement

Ethical review and approval was not required for the study on human participants in accordance with the local legislation and institutional requirements. Written informed consent to participate in this study was provided by the participants' legal guardian/next of kin.

## Author Contributions

AS-P was responsible for the conception and design of the study, collection, and interpretation of clinical data and drafted the manuscript. LO was involved in the collection of clinical data and participated in drafting the manuscript. KJ-P was responsible for the interpretation of radiological data and participated in drafting the manuscript. All authors contributed to the article and approved the submitted version.

## Conflict of Interest

The authors declare that the research was conducted in the absence of any commercial or financial relationships that could be construed as a potential conflict of interest.
